# *Plasmodium falciparum* elimination challenges in a hyperendemic setting, Guéckédou prefecture, Republic of Guinea

**DOI:** 10.1186/1475-2875-11-S1-P97

**Published:** 2012-10-15

**Authors:** Amanda J Tiffany, Esther Sterk, Micaela Serafini, Eric Comte, Melat Halle, Klaudia Porten

**Affiliations:** 1 Epicentre, Paris, France; 2 Médecins Sans Frontières Switzerland, Geneva, Switzerland; 3 Médecins Sans Frontières Switzerland, Conakry, Guinea

## Background

The Republic of Guinea (Guinea) has year round malaria transmission with a seasonal peak corresponding with the rainy season. According to both programmatic and health center data, malaria caused by *Plasmodium falciparum* remains the principle public health problem for the population. In 2010, Médecins Sans Frontières (MSF) implemented an innovative multi-component malaria intervention in Guéckédou prefecture, south-western Guinea. The objective of the intervention is to decrease morbidity, mortality and transmission of malaria by reinforcing health facilities and ensuring access to malaria tests and treatment. The impact of the intervention is measured by a series of cross sectional surveys.

## Materials and methods

In April 2011 the first of seven cross sectional surveys was carried out in four strata; two rural areas and one urban area with MSF intervention and one rural area with limited MSF support. Within each strata probability proportionate to size cluster sampling was used to select clusters. Households were randomly selected in each cluster and all permanent residents invited to participate. Informed consent was obtained from all participants or their guardian. In addition to administering a standardized questionnaire and carrying out a brief clinical exam, a blood slide with both thick and thin films was made and each participant was tested for malaria using SD Bioline HRPII rapid test regardless of their symptoms. All participants who tested RDT positive on the day of the survey were treated with the first line malaria treatment according to the National Malaria Control Protocol or referred to the nearest health facility.

## Results

Over 6,800 people participated in this survey. Children <5 years of age constituted 31% of the survey sample, children 5-14 years of age 27%, and individuals ≥15 years 42%. Women comprised 56.1% of the surveyed population and men 43.9%. A total of 4788 available samples were positive for malaria with microscopy for a point prevalence of 68.6% (n=4788, 95% CI 65.7%-71 .5%) while 61.3% of individuals were identified as positive with RDT (n=4435,95% CI 59.1 %-63.4%). 99.5% of microscopically diagnosed malaria infections were due to *Plasmodium falciparum* infection. Further laboratory quantification revealed that 11.1% of infected individuals had levels of parasitemia within the lower limits of RDT detection, 50-100 parasites/µl and 18.5% of microscopy positive individuals had parasitemia below the RDT detection threshold, ≤50 parasites/µl and were RDT negative.

## Conclusions

Prior to our survey, aside from health facility estimates, the malaria burden in and around Guéckédou was unknown. Laboratory analysis demonstrates that the gold standard, microscopy, remains the most reliable method to detect malaria infections. Additionally, exclusive diagnosis with malaria rapid tests similar to those currently on the market will not identify all parasitaemic individuals. In Guéckédou, a hyper endemic context, 18.5% of infected individuals were not identified when only using RDTs for diagnosis. In areas targeted for elimination where a significant proportion of the population has low level parasitemia the use of rapid diagnostic tests alone is not sufficient to detect and thus eliminate the parasite reservoir.

